# Limited duration of vaccine poliovirus and other enterovirus excretion among human immunodeficiency virus infected children in Kenya

**DOI:** 10.1186/1471-2334-9-136

**Published:** 2009-08-23

**Authors:** Nino Khetsuriani, Rita Helfand, Mark Pallansch, Olen Kew, Ashley Fowlkes, M Steven Oberste, Peter Tukei, Joseph Muli, Ernest Makokha, Howard Gary

**Affiliations:** 1 Centers for Disease Control and Prevention, Atlanta, Georgia, USA; 2 Kenya Medical Research Institute, Nairobi, Kenya

## Abstract

**Background:**

Immunodeficient persons with persistent vaccine-related poliovirus infection may serve as a potential reservoir for reintroduction of polioviruses after wild poliovirus eradication, posing a risk of their further circulation in inadequately immunized populations.

**Methods:**

To estimate the potential for vaccine-related poliovirus persistence among HIV-infected persons, we studied poliovirus excretion following vaccination among children at an orphanage in Kenya. For 12 months after national immunization days, we collected serial stool specimens from orphanage residents aged <5 years at enrollment and recorded their HIV status and demographic, clinical, immunological, and immunization data. To detect and characterize isolated polioviruses and non-polio enteroviruses (NPEV), we used viral culture, typing and intratypic differentiation of isolates by PCR, ELISA, and nucleic acid sequencing. Long-term persistence was defined as shedding for ≥ 6 months.

**Results:**

Twenty-four children (15 HIV-infected, 9 HIV-uninfected) were enrolled, and 255 specimens (170 from HIV-infected, 85 from HIV-uninfected) were collected. All HIV-infected children had mildly or moderately symptomatic HIV-disease and moderate-to-severe immunosuppression. Fifteen participants shed vaccine-related polioviruses, and 22 shed NPEV at some point during the study period. Of 46 poliovirus-positive specimens, 31 were from HIV-infected, and 15 from HIV-uninfected children. No participant shed polioviruses for ≥ 6 months. Genomic sequencing of poliovirus isolates did not reveal any genetic evidence of long-term shedding. There was no long-term shedding of NPEV.

**Conclusion:**

The results indicate that mildly to moderately symptomatic HIV-infected children retain the ability to clear enteroviruses, including vaccine-related poliovirus. Larger studies are needed to confirm and generalize these findings.

## Background

With progress toward eradication of poliovirus circulation in most countries of the world, discussions are underway on vaccination policies in the post-eradication era [1,2]. Following polio eradication, paralytic poliomyelitis from poliovirus will only occur as a result of the continued use of the live oral poliovirus vaccine (OPV). This includes vaccine-associated paralytic poliomyelitis (VAPP), which occurs at a very low rate wherever OPV is used, as well as poliomyelitis associated with vaccine-derived polioviruses (VDPV) [1,3-8]. The potential for VDPV persistence and circulation is one of several critical risks to be considered when developing strategies for stopping polio vaccination after global eradication of wild polioviruses [2].

Immunodeficient persons with persistent vaccine-related poliovirus infection may serve as a potential reservoir for reintroduction of polioviruses into the general population after wild poliovirus eradication, posing a risk of their further circulation in inadequately immunized populations. Chronic enterovirus persistence and increased risk of VAPP among immunodeficient persons with B-cell and combined deficiencies are well documented [9,10]. Chronic VDPV persistence has been documented for persons with certain defects of antibody production [1,2,8], but little is known about poliovirus persistence among human immunodeficiency virus (HIV)-infected persons.

Two studies reported that HIV-positive children were more likely to be infected with enteroviruses than healthy children [11,12], and in one of them, enterovirus excretion by an HIV-positive child for up to 6 months was observed [12]. One study described a case of chronic enterovirus meningoencephalitis in an HIV-infected adult, successfully treated with pleconaril [13]. However, other studies found no evidence of prolonged or more severe enterovirus infections among HIV-infected persons [14,15]. Also, available data do not support the increased risk or severity of wild or vaccine-associated poliomyelitis in HIV-infected individuals. OPV immunization of HIV-infected children results in protective, although somewhat lower, antibody titers compared with HIV-uninfected children, with a comparable low rate of adverse events in both groups [16,17]. OPV is accepted by the World Health Organization (WHO) as safe and immunogenic in HIV-infected persons [18,19] and is widely used in countries with large HIV-infected populations. Only two cases of VAPP in HIV-infected patients have been reported [20,21]. However, because of the large numbers of HIV-infected persons in developing countries exposed to OPV, and the serious public health implications of potential long-term VPDV persistence, the risk of the vaccine-related poliovirus persistence among HIV-infected persons needs to be evaluated.

To estimate the potential for vaccine-related poliovirus persistence among immunodeficient persons, we conducted this study of HIV-infected children in Kenya.

## Methods

### Study population

We conducted a prospective study of persistence of vaccine-related poliovirus and non-polio enteroviruses (NPEV) among children who lived in a small orphanage for HIV-infected children in Nairobi, Kenya. The orphanage (capacity, 40–45 children) admits only children aged <5 years who test seropositive for HIV. At the orphanage, children receive adequate nutrition and medical care, including immunizations. Anti-retroviral treatment was not routinely available at the time the study was conducted.

By reviewing medical records at the orphanage, we identified each child's HIV status and obtained demographic, clinical, and immunization data. Final HIV infection status of the participants was assigned at the time of follow-up in November-December 2000, when all the study participants were aged >18 months. Children who had reverted to seronegative were classified as HIV-uninfected and served as the control group. Clinical categories and immunologic status of HIV-infected children were assigned according to the 1994 Revised Classification System for HIV Infection in Children <13 years of age from the Centers for Disease Control and Prevention (CDC) [22]. The age-specific criteria of the degree of immunosuppression defined by CDC are the following: 1) no evidence of immunosuppression – age <12 months: CD4+ count ≥ 1500, and/or CD4% ≥ 25%; age 1–5 years: CD4+ count ≥ 1000 and/or CD4+% ≥ 25%; 2) moderate immunosuppression – age <12 months: CD4+ count 750–1499, and/or CD4% 15–24%; age 1–5 years: CD4+ count 500–999 and/or CD4+% 15–24%; 3) severe immunosuppression – age <12 months: CD4+ count <750, and/or CD4% <15%; age 1–5 years: CD4+ count <500 and/or CD4+% 15% [22]. The dates of the last OPV exposure were defined as the date of the second round of the national immunisation days (NIDs) for those who did not receive routine OPV doses after study enrollment, or as the most recent immunization date prior to each specimen collection for children who received routine OPV after study enrollment.

### Specimen collection

We collected serial stool specimens for 12 months after the 1998 NIDs (during September 1998–Agust, 1999) from children residing at the orphanage who were <5 years of age at the time of enrollment. The intervals between specimen collections ranged from 5 days to 4 weeks.

### Laboratory analysis

Stool specimens were stored at -20°C at the Kenya Medical Research Institute (KEMRI), Nairobi, and transported on dry ice to the CDC Polio and Picornavirus Laboratory (Atlanta, GA) for testing. Viral culture, typing and intratypic differentiation of isolates by PCR and ELISA [23,24], and nucleic acid sequencing of the VP1 gene (~900 nucleotides) [7] were used to determine the type of poliovirus and the degree of its relatedness to the prototype Sabin strains. Selected poliovirus isolates were screened by PCR to detect recombination with other polioviruses or NPEV, and some isolates underwent complete genomic sequencing [25,26]. Phylogenetic relationships among the isolates were inferred by using the neighbor-joining algorithm (PHYLIP: Phylogeny Inference Package, version 3.57, University of Washington, Seattle, WA). NPEV detected by virus isolation were identified by sequencing a portion of the VP1 gene [27,28]. Long-term persistence was defined as shedding of vaccine-related poliovirus or the same serotype of NPEV for ≥ 6 months.

Blood specimens for lymphocyte subset analysis were obtained from each participant at least once during the study period and were tested for CD3, CD4, and CD8 antigens at the HIV laboratory, KEMRI, by flow cytometric analysis using monoclonal antibodies.

The study protocol was approved by the Institutional Review Boards of CDC and KEMRI.

## Results

### Study enrollment

We enrolled 24 children (Table 1 and Additional file 1: Table S1), all of whom were HIV-seropositive at the time of their admission to the orphanage. Fourteen children were enrolled in September, 1998, immediately after the NIDs, and 10 children who were admitted to the orphanage after the NIDs, were enrolled 3 to 6 months later. In accordance with CDC criteria, 15 of these were confirmed to be HIV-infected, while 9 children reverted to seronegative status and were therefore reclassified as HIV-uninfected. Median age at enrollment was 30 months (range, 5–52) for HIV-infected children and 12 months (range, 4–30) for those in the HIV-uninfected group. All HIV-infected children had clinically manifested disease ranging from mildly symptomatic (category A) (3 children) to moderately symptomatic (category B) (12 children) [22]. All 15 HIV-infected children were immunocompromised: eight had moderate and seven had severe immunosuppression according to CDC age-specific criteria [22]. None of the HIV-uninfected children in the study had any clinical or immunologic evidence of immunosuppression. Two HIV-infected children died from conditions related to their HIV infection in 2000, prior to the follow-up conducted later that year.

**Table 1:** The results of viral culture of stool specimens from children at an orphanage in Kenya, by HIV-status

Parameters	Total, n (%)	HIV(+), n (%)	HIV(-), n (%)
**Children in the study**	24 (100.0)	15 (100.0)	9 (100.0)
Children with ≥ 1 specimen positive for:			
Polioviruses	15 (62.5)	10 (66.7)	5 (55.6)
NPEV^a^	22 (91.7)	11 (93.3)	8 (88.9)
Other viruses^b^	5 (20.8)	2 (13.3)	3 (33.3)
			
**Specimens**	255 (100.0)	170 (100.0)	85 (100.0)
Specimens positive for:			
Any virus	126 (49.4)	78 (45.9)	48 (56.25)
Polioviruses	46 (18.0)	31 (18.2)	15 (17.26)
NPEV	77 (30.2)	48 (28.2)	29 (34.1)
Other viruses	8 (3.1)	3 (1.78)	5 (5.79)
Specimens with no viruses isolated	129 (50.6)	92 (54.1)	37 (43.5)

^a ^NPEV, non-polio enteroviruses. NPEV serotypes included coxsackieviruses A4 (n = 18), A16 (n = 1), and A24 (n = 7); echoviruses 2 (n = 1), 4 (n = 15), 12 (n = 3), 17 (n = 6), and 25 (n = 10); and enteroviruses 80 (n = 12) and 99 (n = 4).

^b ^The viruses other than enteroviruses have not been further characterized.

There were no statistically significant differences between HIV-positive and HIV-negative groups.

Routine OPV immunization history was documented for 13 children. All had received three or four OPV doses. For two additional children, having received 3 doses of OPV was noted but vaccination dates were not given. Five (2 HIV-infected, 3 HIV-uninfected) children received routine doses of OPV vaccine during the enrollment period. As reported by the orphanage staff, all study participants who resided at the orphanage as of August, 1998, received two supplemental doses of OPV during NIDs (on August 8 and September 12, 1998). However, in accordance with local practice and WHO guidelines, these doses were not documented in their medical records. The medical record review showed that no illnesses were recorded for any of the children during the period immediately preceding the immunization rounds that could potentially have been a contraindication to the receipt of supplemental doses of OPV during NIDs.

A total of 255 stool specimens were obtained, 170 from HIV-infected children (median 13 per child, range 6–15) and 85 from HIV-uninfected children (median 8 per child, range 7–12). Specimens were collected for a median of 11 months (range, 5–12) for HIV-infected children and 8 months (range, 7–12) for the HIV-uninfected children.

### Virus isolation

All children in the study had positive viral culture results at some point during the study period. Of the 126 positive specimens, 78 were from HIV-infected and 48 were from HIV-uninfected children. The detailed results of viral culture of stool specimens by HIV status are presented in Table 1 and Additional file 1: Table S1.

All poliovirus isolates from study participants were vaccine-related. Type 1 poliovirus was detected in 26 specimens (including 18 specimens from HIV-infected children), type 2 in 12 specimens (7 from HIV-infected children), and type 3 in 14 specimens (8 from HIV-infected). More than one poliovirus type was present in five specimens (2 from HIV-infected). Fourteen children had more than one specimen positive for poliovirus (9 HIV-infected and 5 HIV-uninfected). There were no significant differences in the distribution of poliovirus serotypes between the HIV-infected and HIV-uninfected groups.

NPEVs representing 10 different serotypes were detected in 77 specimens (including 48 specimens from HIV-infected children) (Table 1 and Additional file 1: Table S1; GenBank accession numbers GQ176161–GQ176237). The most frequently detected serotypes were coxsackievirus A4 and echovirus 4 (detected in 18 and 15 specimens, respectively); four other serotypes were detected in five or more specimens. Twenty NPEV-positive children (12 HIV-infected and 8 HIV-uninfected) had more than one specimen positive for NPEV. The number of NPEV-positive specimens per child throughout the entire study period ranged from 1 to 6 per child (median, 4), and each NPEV-positive child had up to six different serotypes isolated at different times. There were multiple introductions of NPEV into the orphanage with subsequent apparent spread of the same serotype to other children. Of the 77 NPEV detections, 65 were associated with new infections (42 in HIV-infected and 23 in HIV-uninfected children), and 12 (5 in HIV-infected and 7 in HIV-uninfected children) reflected continuous shedding after the initial infection. There were no significant differences in prevalence of NPEV shedding by HIV status (Chi-square, p > 0.05; Table 1) or by age (29.8% for children who were <24 months-old at the time of specimen collection versus 29.3% for children who were >24 months of age; Chi-square, p > 0.05).

### Duration of shedding

There was no virologic evidence of prolonged poliovirus or NPEV shedding in study participants. Polioviruses were not detected in any of the specimens obtained ≥ 6 months after the last OPV dose, and none of the children had more than two consecutive specimens positive for the same NPEV serotype. The median interval between the first and last poliovirus-positive specimen was 50 days (range, 48–142) for the seven HIV-infected and 57 days (range, 34–115) for the three HIV-uninfected children. Children known to have received routine doses of OPV during the study period or who had only one poliovirus-positive sample were excluded from this analysis.

The HIV-infected child with the 142-day interval between the first and last poliovirus-positive specimens (participant F) had 14 specimens collected. Vaccine-related poliovirus type 1 was detected in three of the four specimens obtained between days 3 and 58 after the second round of NIDs. After that time, the child stopped shedding type 1 virus and three of the four specimens collected between days 82 and 142 were positive for vaccine-related poliovirus type 2. The subsequent six specimens were negative. This child, aged 45 months at enrollment, was severely immunocompromised [CD4+ cells ranged between 298 (16%) and 548 (18%)] and had clinical category B AIDS. She had received three doses of OPV in 1996, two years prior to the enrolment in the present study (Additional file 1: Table S1).

The HIV-uninfected child who shed polioviruses over 115 days (participant J) was admitted to the orphanage after the 1998 NIDs. She was enrolled into the study at the age of 4 months and had seven stool specimens collected over 6 months. The first specimen was positive for all three types of polioviruses; the second, obtained 27 days later was negative; the third, collected 58 days after the first specimen, contained polio vaccine virus type 1; two subsequent specimens, collected at 87 and 115 days, contained polio vaccine virus type 3. The last two specimens were positive for two different NPEV (enterovirus 80 and coxsackievirus A4). The child's medical record noted she that had received 3 OPV doses, but the dates were not recorded (Additional file 1: Table S1).

### Genetic characterization of poliovirus isolates

All of the poliovirus isolates appeared "Sabin-like" in the ELISA intratypic differentiation tests, indicating the lack of any major antigenic changes. The viruses were sequenced to measure the degree of genetic relatedness of isolated polioviruses compared to the prototype Sabin strains. We sequenced the VP1 gene of isolates (n = 30) from children with unknown vaccination history, as well as selected isolates from other participants. Of these, 18 isolates were type 1 (11 from HIV-infected, 7 from HIV-uninfected participants), seven were type 2 (4 from HIV-infected, 3 from HIV-uninfected), and six were type 3 (3 from HIV-infected, 1 from HIV-uninfected).

For type 1 polioviruses, genomic sequences of the VP1 gene were identical or closely related to the Sabin vaccine strains for 15 isolates, with 0 to 4 nucleotide substitutions over the VP1 region. However, an increasing degree of divergence was observed for three serial type 1 isolates obtained from one child (participant A) on days 37, 51, and 87 after the second round of NIDs, with 0.33%, 0.66%, and 0.99% divergence in VP1 sequence from the prototype Sabin strain, respectively. The degree of divergence in the last isolate approached the 1% cut-off level defining VDPV. Complete genomic sequences of these three isolates, however, were only 0.21%, 0.24%, and 0.42% divergent from the type 1 Sabin strain, respectively. The PCR assays for recombination performed on these isolates did not reveal any evidence of recombination with other vaccine-related or wild polioviruses or NPEV.

The source of these isolates (participant A) was an HIV-infected girl, aged 52 months at enrollment, with unknown history of previous OPV vaccination (Additional file 1: Table S1). Clinically, she had category B (moderately symptomatic) AIDS. Her medical history prior to and during the study period included multiple opportunistic infections, pulmonary tuberculosis, convulsions and petit mal seizures, and episodes of malaria. Her CD4+ lymphocyte count was 746 (12%) 4 months after enrollment, and 606 (13%) 9 months after enrollment. The child remained at the orphanage at follow-up in late 2000. Of her 13 stool specimens collected over 10 months, four were positive for type 1 polio vaccine virus, four were positive for four different NPEV (coxsackievirus A4, echoviruses 4 and 17, and enterovirus 80), and five were negative.

**Figure 1:**
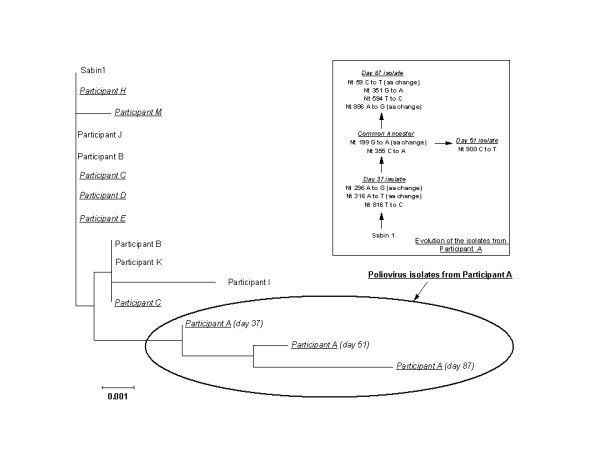
**Phylogenetic tree of poliovirus type 1 isolates from study participants**. Isolates from HIV-infected children are shown in *italic *font. The inferred evolution of the isolates from participant A is shown in the insert. Nt, nucleotide; aa, amino acid.

In the phylogenetic tree of the type 1 polioviruses isolated from the study participants based on the VP1 gene sequences (Figure 1), the isolates from participant A clustered together and were distinct from the prototype Sabin strain and from the polioviruses isolated from other study children. All sequenced type 2 and type 3 poliovirus isolates from study participants were closely related to the prototype Sabin strains, differing by only one or two nucleotide substitutions. The degree of divergence in VP1 gene sequences ranged from 0 to 0.22% for type 2 isolates and from 0 to 0.36% for type 3 isolates. The type 2 poliovirus isolate from the last positive specimen of participant F was only 0.22% divergent from the prototype Sabin strain.

## Discussion

In this study, we found no clinical evidence of long-term (≥ 6 months based on the date of the last known dose of OPV) persistence of vaccine-related polioviruses or NPEV among mildly to moderately symptomatic HIV-infected children in Kenya. In all cases, the poliovirus infections were cleared during the observation period. The overall patterns of excretion did not differ by HIV status.

There are very few reports exploring poliovirus excretion by HIV-infected persons. These studies did not reveal prolonged excretion of vaccine-related poliovirus by HIV-infected adults [15,29,30] or children [30]. In one study [31] three HIV-infected children from South Africa were found to be shedding vaccine-related polioviruses in specimens obtained between 15 and 42 months after the last known dose of OPV, suggesting possible long-term persistence. However, subsequent molecular studies showed that for all three cases, the divergence of nucleotide sequences in the VP1 region was 0.3% to 0.6% [32]. This close genetic relatedness to prototype Sabin strains is not consistent with prolonged persistence and suggests relatively recent re-infection from repeated exposure to vaccine-related poliovirus in a country with widespread routine OPV use. In another study, stool specimens from two of the 11 participants who had received OPV in the past were positive for polioviruses [12]. However, the duration of poliovirus persistence in these cases could not be estimated and no further characterization of the isolates was attempted.

Unlike other studies which examined point prevalence of vaccine-related poliovirus shedding in HIV-infected individuals, the prospective design and the availability of serial specimens in this study allowed us to evaluate the duration of viral shedding after each episode of infection and to monitor genetic changes of poliovirus isolates over time. In our study, the interval between the first and the last poliovirus-positive specimens exceeded the expected range of 2–3 months [18,33,34] for two participants. However, none of these children shed divergent strains or were true long-term shedders. Repeated infection from exposure to other vaccinees at the orphanage may have occurred in participant F, who began excreting poliovirus type 2 after initially shedding poliovirus type 1. Close genetic relatedness of these viruses to the prototype Sabin strains suggests that they could not have originated from routine OPV doses received two years previously. In case of participant J, the dates when OPV doses were given were unavailable, but the age (4 months), recent admission to the orphanage, the mention of having received 3 OPV doses in medical record, and the pattern of shedding suggest that this child likely had received one or more doses of OPV immediately before or during the study enrolment period. The very close genetic relatedness of the isolates from this child to the prototype vaccine strains also supports this suggestion.

In all but one instance, genetic characterization of polioviruses isolated from the study participants were very closely related to the prototype Sabin strains, with <0.5% difference in VP1 sequence, consistent with the typical short periods of replication. This makes us confident, that even if in some cases the infection persisted from previous exposures, it is unlikely that the actual duration of persistence would be substantially longer. Type 1 isolates from one child (participant A) showed a higher degree of divergence in the VP1 gene. However, this child was not a prolonged shedder, and the divergence from the prototype strain did not result from prolonged circulation. We were able to observe continuous pattern of relatively fast accumulation of genetic changes (rate, about 0.33% nucleotide substitutions per month) in this strain, beginning from 0.33% divergence (3 substitutions) and approaching with 9 substitutions (0.99% divergence) at 3 months after exposure, the 1% cut-off currently defining a strain as a VDPV (annualized rate of accumulating substitutions, ~4%). In addition the virus shedding stopped in specimens obtained after day 87. The degree of divergence based on the complete genomic sequences of these isolates was considerably lower (0.42% by day 87, corresponding to an annualized rate of ~1.7%) than that based on the sequences of VP1 gene only. The average rate of ~1% per year for total nucleotide substitutions for polioviruses in the VP1 gene region is well established [35,36]. However, with relatively short observation periods (e.g., a few months), confidence intervals around point estimates for the duration of persistence based on the degree of divergence can be very wide because of the stochastic nature of mutations. The higher rate of change can also be attributed to the high proportion of non-synonymous changes seen in this case (5/9; Figure 1), which may be related to the immunological status of this child. It is important to note that despite the frequent spread of NPEV among study participants, there was no evidence of poliovirus transmission from participant A to other children at the orphanage, as type 1 poliovirus isolates from other children were genetically distinct from her isolates (Figure 1). The lack of evidence of recombination with other polioviruses or NPEV is also compatible with an observed relatively short period of replication in this case.

The results of the present study indicate that the ability to clear OPV and other enteroviruses is preserved in mildly to moderately symptomatic HIV-infected children. The lack of evidence for chronic persistence of NPEV in the study population further strengthens this conclusion. A number of factors could potentially influence the course of poliovirus infection in an HIV-infected host, including the degree of immunosuppression, previous OPV vaccination history, and patient age. However, the small sample size, incomplete OPV exposure data for some children, and limited clinical information prevented more detailed analysis of these variables. As none of the HIV-infected children in the study had category C (severely symptomatic) AIDS, we were unable to observe the subset of patients with highly advanced HIV disease, who may be at a higher risk of developing secondary defects of humoral immunity resulting from deep T-cell impairment. However, the short life expectancy of category C patients, especially in the absence of specific antiretroviral treatment [37], would reduce their overall potential for further transmission of VDPVs, even in the context of persistent infection. Also, because of the relatively high standard of care and nutrition at the orphanage, the study participants may not be completely representative of the general population of HIV-infected children in Kenya and in other developing countries.

## Conclusion

In this study, mildly to moderately symptomatic HIV-infected children retained the ability to clear enteroviruses, including vaccine-related polioviruses. These results are reassuring as to the limited replication of poliovirus in HIV-infected children. To fully characterize the patterns of VDPV excretion among HIV-infected persons and to quantify the risk of long-term VDPV replication in this group, larger studies that cover the full range of HIV disease are needed.

## Competing interests

The authors declare that they have no competing interests.

## Authors' contributions

NK participated in the study conception and design, data collection, data analysis, overall coordination, drafted the manuscript; RH participated in the study conception and design, data collection, data analysis, overall coordination, write-up; MP participated in the study conception and design and laboratory analysis (virology testing for polioviruses); OK participated in the study conception and design and laboratory analysis (molecular studies of polioviruses); AF – participated in the study data acquisition and data analysis; MSO – participated in laboratory analysis (virologic and molecular studies of NPEV); PT – participated in the study conception and design, coordinated data collection; JM – participated in the data collection; EM – participated in laboratory analysis (HIV testing, CD4/CD8 counts); HG – participated in the study conception and design and data collection. All authors read and approved the final manuscript.

## Pre-publication history

The pre-publication history for this paper can be accessed here:

http://www.biomedcentral.com/1471-2334/9/136/prepub

## Supplementary Material

Additional file 1 **Supplementary table S1**. Study participants age, HIV infection status, routine OPV immunization history and results of stool testing. Click here for file
