# Supplementary material for: Limited duration of vaccine poliovirus and other enterovirus excretion among human immunodeficiency virus infected children in Kenya
Source: BMC Infect Dis. 2009 Aug 23;9:136. doi: 10.1186/1471-2334-9-136 (PMC2739212; doi:10.1186/1471-2334-9-136)
Supplement: Additional file 1 — Supplementary table S1. Study participants age, HIV infection status, routine OPV immunization history and results of stool testing. [file 1471-2334-9-136-S1.pdf]

Table S1. Study participants' age, HIV infection status, routine OPV immunization history and results of stool testing

| Participant ID/age, months | HIV infection status | Routine OPV doses | Specimen Date | Specimen | Viral culture result | Genotype | Nucleotide changes, % | NPEV serotype |
|---|---|---|---|---|---|---|---|---|
| A/52 | Inf. | Unk. | 9/16/98 | ST1 | NPEV | | | EV80 |
| | | | 10/19/98 | ST2 | PV1 | SABIN | 0.33 | |
| | | | 11/2/98 | ST3 | PV1 | SABIN | 0.66 | |
| | | | 11/9/98 | ST4 | NEGATIVE | | | |
| | | | 12/3/98 | ST5 | PV1 | SABIN | | |
| | | | 12/8/98 | ST6 | PV1 | SABIN | 0.99 | |
| | | | 1/4/99 | ST7 | NEGATIVE | | | |
| | | | 2/4/99 | ST8 | NEGATIVE | | | |
| | | | 3/5/99 | ST9 | NEGATIVE | | | |
| | | | 4/2/99 | ST10 | NEGATIVE | | | |
| | | | 4/30/99 | ST11 | NPEV | | | CA4 |
| | | | 6/25/99 | ST12 | NPEV | | | E17 |
| | | | 7/22/99 | ST13 | NPEV | | | E4 |
| C/27 | Inf. (died in 2000) | 3 doses, in 1996 | 9/16/98 | ST1 | PV1 | SABIN | 0 | |
| | | | 10/19/98 | ST2 | PV1 | SABIN | 0.11 | |
| | | | 11/2/98 | ST3 | PV2 | SABIN | 0.11 | |
| | | | 11/9/98 | ST4 | PV2 | SABIN | 0.11 | |
| | | | 12/3/98 | ST5 | NEGATIVE | | | |
| | | | 12/8/98 | ST6 | NEGATIVE | | | |
| | | | 1/4/99 | ST7 | NEGATIVE | | | |
| | | | 2/4/99 | ST8 | NEGATIVE | | | |
| | | | 3/5/99 | ST9 | NEGATIVE | | | |
| | | | 4/2/99 | ST10 | NPEV | | | E12 |
| | | | 4/30/99 | ST11 | NEGATIVE | | | |
| | | | 5/28/99 | ST12 | NEGATIVE | | | |
| | | | 6/25/99 | ST13 | NPEV | | | E17 |
| | | | 7/22/99 | ST14 | NPEV | | | E4 |
| | | | 8/18/99 | ST15 | NPEV | | | E4 |
| D/27 | Inf. | 3 doses, in 1996-1997 | 9/16/98 | ST1 | PV1 | SABIN | 0 | |
| | | | 10/19/98 | ST2 | NEGATIVE | | | |
| | | | 11/2/98 | ST3 | PV2 | SABIN | 0.11 | |
| | | | 11/9/98 | ST4 | NEGATIVE | | | |
| | | | 12/3/98 | ST5 | NEV | | | |
| | | | 12/8/98 | ST6 | NEGATIVE | | | |
| | | | 4/1/99 | ST7 | NEGATIVE | | | |
| | | | 2/4/99 | ST8 | NPEV | | | E25 |
| | | | 3/5/99 | ST9 | NEGATIVE | | | |
| | | | 4/2/99 | ST10 | NPEV | | | E12 |
| | | | 4/30/99 | ST11 | NPEV | | | CA4 |

| Participant ID/age, months | HIV infection status | Routine OPV doses | Specimen Date | Specimen | Viral culture result | Genotype | Nucleotide changes, % | NPEV serotype |
|---|---|---|---|---|---|---|---|---|
| | | | 5/28/99 | ST12 | NPEV | | | CA4 |
| | | | 6/25/99 | ST13 | NEGATIVE | | | |
| | | | 7/22/99 | ST14 | NPEV | | | E4 |
| | | | 8/18/99 | ST15 | NEGATIVE | | | |
| E/33 | Inf. | Unk. | 9/16/98 | ST1 | NEGATIVE | | | |
| | | | 10/19/98 | ST2 | PV1 | SABIN | 0 | |
| | | | 11/2/98 | ST3 | NEGATIVE | | | |
| | | | 11/9/98 | ST4 | NEGATIVE | | | |
| | | | 12/3/98 | ST5 | NEGATIVE | | | |
| | | | 12/8/99 | ST6 | NEGATIVE | | | |
| | | | 2/4/99 | ST7 | NEGATIVE | | | |
| | | | 3/5/99 | ST8 | NEGATIVE | | | |
| | | | 4/2/99 | ST9 | NEGATIVE | | | |
| | | | 4/30/99 | ST10 | NEGATIVE | | | |
| | | | 5/28/99 | ST11 | NEGATIVE | | | |
| | | | 6/25/99 | ST12 | NPEV | | | E17 |
| | | | 7/22/99 | ST13 | NPEV | | | E4 |
| | | | 8/18/99 | ST14 | NEGATIVE | | | |
| F/45 | Inf. | 3 doses, in 1996 | 9/16/98 | ST1 | PV1 | SABIN | | |
| | | | 10/19/98 | ST2 | NEGATIVE | | | |
| | | | 11/2/98 | ST3 | PV1 | SABIN | | |
| | | | 11/9/98 | ST4 | PV1 | SABIN | 0 | |
| | | | 12/3/98 | ST5 | PV2 | SABIN | | |
| | | | 12/8/98 | ST6 | PV2 | SABIN | | |
| | | | 1/4/99 | ST7 | NEGATIVE | | | |
| | | | 2/4/99 | ST8 | PV2 | SABIN | 0.22 | |
| | | | 3/5/99 | ST9 | NEGATIVE | | | |
| | | | 4/2/99 | ST10 | NEGATIVE | | | |
| | | | 4/30/99 | ST11 | NEGATIVE | | | |
| | | | 5/28/99 | ST12 | NEGATIVE | | | |
| | | | 6/25/99 | ST13 | NEGATIVE | | | |
| | | | 7/22/99 | ST14 | NEGATIVE | | | |
| G/33 | Inf. | 4 doses, in 1996-1997 | 9/16/98 | ST1 | NEGATIVE | | | |
| | | | 10/19/98 | ST2 | NEGATIVE | | | |
| | | | 11/2/98 | ST3 | NEGATIVE | | | |
| | | | 11/9/98 | ST4 | NEGATIVE | | | |
| | | | 12/3/98 | ST5 | NEGATIVE | | | |
| | | | 1/4/99 | ST6 | NEGATIVE | | | |
| | | | 2/4/99 | ST7 | NEGATIVE | | | |
| | | | 3/5/99 | ST8 | NEGATIVE | | | |
| | | | 4/2/99 | ST9 | NEGATIVE | | | |
| | | | 4/30/99 | ST10 | NPEV | | | CA4 |
| | | | 5/28/99 | ST11 | NPEV | | | CA4 |

| Participant ID/age, months | HIV infection status | Routine OPV doses | Specimen Date | Specimen | Viral culture result | Genotype | Nucleotide changes, % | NPEV serotype |
|---|---|---|---|---|---|---|---|---|
| | | | 6/25/99 | ST12 | NEGATIVE | | | |
| | | | 7/22/99 | ST13 | NPEV | | | E4 |
| H/20 | Inf. (died in 2000) | Unk. | 9/16/98 | ST1 | PV1,3 | SABIN | 0 (PV1), 0.32 (PV3) | |
| | | | 10/19/98 | ST2 | NPEV | | | EV80 |
| | | | 11/2/98 | ST3 | PV1 | SABIN | | |
| | | | 11/9/98 | ST4 | NEGATIVE | | | |
| | | | 12/3/98 | ST5 | NEGATIVE | | | |
| | | | 12/8/98 | ST6 | NEGATIVE | | | |
| | | | 1/4/99 | ST7 | NEGATIVE | | | |
| | | | 2/4/99 | ST8 | NPEV | | | E25 |
| | | | 3/5/99 | ST9 | NPEV | | | E25 |
| | | | 4/2/99 | ST10 | NEGATIVE | | | |
| | | | 4/30/99 | ST11 | NPEV | | | CA4 |
| | | | 5/28/99 | ST12 | NEGATIVE | | | |
| | | | 6/25/99 | ST13 | NPEV | | | CA4 |
| | | | 7/22/99 | ST14 | NPEV | | | E4 |
| | | | 8/18/99 | ST15 | NPEV | | | E4 |
| L/5 | Inf. | 3 doses, shortly before and during enrolment (1/13/99, 2/10/99, 3/10/99) | 2/4/99 | ST1 | PV3 | SABIN | | |
| | | | 3/5/99 | ST2 | PV3 | SABIN | | |
| | | | 4/2/99 | ST3 | PV1,3 | SABIN | 0 (PV1), 0.11 (PV3) | |
| | | | 4/30/99 | ST4 | NPEV | | | CA4 |
| | | | 5/28/99 | ST5 | NEGATIVE | | | |
| | | | 6/25/99 | ST6 | NEGATIVE | | | |
| | | | 7/22/99 | ST7 | NEGATIVE | | | |
| | | | 8/18/99 | ST8 | NPEV | | | E4 |
| M/34 | Inf. | 3 doses, dates unk. | 2/4/99 | ST1 | NEGATIVE | | | |
| | | | 3/5/99 | ST2 | NEGATIVE | | | |
| | | | 4/2/99 | ST3 | PV1 | SABIN | 0.11 | |
| | | | 4/30/99 | ST4 | NPEV | | | EV80 |
| | | | 5/28/99 | ST5 | NPEV | | | CA4 |
| | | | 6/25/99 | ST6 | NEGATIVE | | | |
| | | | 7/22/99 | ST7 | NEGATIVE | | | |
| | | | 8/18/99 | ST8 | NPEV | | | E4 |
| N/50 | Inf. | Unk. | 9/16/98 | ST1 | PV1 | SABIN | | |
| | | | 10/19/98 | ST2 | NEGATIVE | | | |
| | | | 11/2/98 | ST3 | PV3 | SABIN | | |
| | | | 11/9/98 | ST4 | PV3 | SABIN | | |
| | | | 12/3/98 | ST5 | PV3 | SABIN | 0.32 | |
| | | | 12/8/98 | ST6 | PV3 | SABIN | | |
| | | | 1/4/99 | ST7 | NEGATIVE | | | |
| | | | 2/4/99 | ST8 | NEGATIVE | | | |
| | | | 3/5/99 | ST9 | NEGATIVE | | | |

| Participant ID/age, months | HIV infection status | Routine OPV doses | Specimen Date | Specimen | Viral culture result | Genotype | Nucleotide changes, % | NPEV serotype |
|---|---|---|---|---|---|---|---|---|
| | | | 4/2/99 | ST10 | NEGATIVE | | | |
| | | | 4/30/99 | ST11 | NPEV | | | CA4 |
| | | | 5/28/99 | ST12 | NEGATIVE | | | |
| | | | 6/25/99 | ST13 | NEGATIVE | | | |
| | | | 7/22/99 | ST14 | NEGATIVE | | | |
| O/30 | Inf. | 3 doses, during enrolment (10/14/98, 11/11/98, 12/9/98) | 9/16/98 | ST1 | PV1 | SABIN | | |
| | | | 10/19/98 | ST2 | PV1 | SABIN | | |
| | | | 11/2/98 | ST3 | PV2 | SABIN | | |
| | | | 11/9/98 | ST4 | NPEV | | | E25 |
| | | | 12/3/98 | ST5 | NEGATIVE | | | |
| | | | 12/8/98 | ST6 | NEGATIVE | | | |
| | | | 1/4/99 | ST7 | NEGATIVE | | | |
| | | | 2/4/99 | ST8 | NEGATIVE | | | |
| | | | 3/5/99 | ST9 | NPEV | | | E25 |
| | | | 4/2/99 | ST10 | NPEV | | | CA4 |
| | | | 4/30/99 | ST11 | NPEV | | | EV80 |
| | | | 5/28/99 | ST12 | NPEV | | | CA24 |
| | | | 6/25/99 | ST13 | NEGATIVE | | | |
| | | | 7/22/99 | ST14 | NEGATIVE | | | |
| | | | 8/18/99 | ST15 | NPEV | | | EV80 |
| P/44 | Inf. | 3 doses, in 1995-1996 | 4/2/99 | ST1 | NPEV | | | EV99 |
| | | | 4/30/99 | ST2 | NPEV | | | Unresolved mixture |
| | | | 5/28/99 | ST3 | NPEV | | | EV80 |
| | | | 6/25/99 | ST4 | NEGATIVE | | | |
| | | | 7/22/99 | ST5 | NEGATIVE | | | |
| | | | 8/18/99 | ST6 | NPEV | | | CA24 |
| V/5 | Inf. | 3 doses, during enrolment (3/10/99, 5/5/99, 6/23/99) | 2/4/99 | ST1 | NEV | | | |
| | | | 3/5/99 | ST2 | NEV | | | |
| | | | 4/2/99 | ST3 | NEGATIVE | | | |
| | | | 4/30/99 | ST4 | NPEV | | | CA4 |
| | | | 5/28/99 | ST5 | NEGATIVE | | | |
| | | | 6/25/99 | ST6 | NEGATIVE | | | |
| | | | 7/22/99 | ST7 | NEGATIVE | | | |
| | | | 8/18/99 | ST8 | NEGATIVE | | | |
| W/17 | Inf. | Unk. | 4/2/99 | ST1 | NPEV | | | CA24 |
| | | | 4/30/99 | ST2 | NPEV | | | EV80 |
| | | | 5/28/99 | ST3 | NEGATIVE | | | |
| | | | 6/25/99 | ST4 | NEGATIVE | | | |
| | | | 7/22/99 | ST5 | NPEV | | | EV80 |
| | | | 8/18/99 | ST6 | NEGATIVE | | | |
| X/22 | Inf. | 3 doses, in 1997 | 4/2/99 | ST1 | NPEV | | | CA4 |

| Participant ID/age, months | HIV infection status | Routine OPV doses | Specimen Date | Specimen | Viral culture result | Genotype | Nucleotide changes, % | NPEV serotype |
|---|---|---|---|---|---|---|---|---|
| | | | 4/30/99 | ST2 | NPEV | | | CA4, EV80 |
| | | | 5/28/99 | ST3 | NEGATIVE | | | |
| | | | 6/25/99 | ST4 | NEGATIVE | | | |
| | | | 7/22/99 | ST5 | NPEV | | | E4 |
| | | | 8/18/99 | ST6 | NEGATIVE | | | |
| B/19 | Uninf. | Unk. | 9/16/98 | ST1 | PV1 | SABIN | 0 | |
| | | | 10/19/98 | ST2 | PV1 | SABIN | 0.11 | |
| | | | 11/2/98 | ST3 | NEGATIVE | | | |
| | | | 11/9/98 | ST4 | NEGATIVE | | | |
| | | | 12/3/98 | ST5 | NEGATIVE | | | |
| | | | 12/8/98 | ST6 | NEGATIVE | | | |
| | | | 1/4/99 | ST7 | NEGATIVE | | | |
| | | | 2/4/99 | ST8 | NEGATIVE | | | |
| I/30 | Uninf | 3 doses, shortly before and during enrolment (9/2/98, 10/14/98, 11/11/98) | 9/16/98 | ST1 | PV3 | SABIN | 0 | |
| | | | 10/19/98 | ST2 | NPEV | | | EV80 |
| | | | 11/2/98 | ST3 | PV1,2 | SABIN | 0 (PV1), 0 (PV2) | |
| | | | 11/9/98 | ST4 | PV1,2 | SABIN | | |
| | | | 12/3/98 | ST5 | PV1 | SABIN | 0.44 | |
| | | | 12/8/98 | ST6 | NPEV | | | EV99 |
| | | | 1/4/99 | ST7 | PV3 | SABIN | | |
| | | | 2/4/99 | ST8 | NEGATIVE | | | |
| | | | 3/5/99 | ST9 | NEGATIVE | | | |
| | | | 4/2/99 | ST10 | NPEV | | | E12 |
| | | | 4/30/99 | ST11 | NPEV | | | CA4 |
| | | | 5/20/99 | ST12 | NPEV | | | CA4 |
| | | | 6/25/99 | ST13 | NPEV | | | E17 |
| | | | 7/22/99 | ST14 | NPEV | | | E4 |
| | | | 8/18/99 | ST15 | NPEV | | | E4 |
| J/4 | Uninf | 3 doses, dates unk. | 12/8/98 | ST1 | PV1,2,3 | SABIN | 0 (PV1), 0 (PV2), 0.36 (PV3) | |
| | | | 1/4/99 | ST2 | NEGATIVE | | | |
| | | | 2/4/99 | ST3 | PV1 | SABIN | 0.33 | |
| | | | 3/5/99 | ST4 | PV3 | SABIN | | |
| | | | 4/2/99 | ST5 | PV3 | SABIN | 0 | |
| | | | 4/30/99 | ST6 | NPEV | | | EV80 |
| | | | 5/28/99 | ST7 | NPEV | | | CA4 |
| K/4 | Uninf | Unk. | 12/8/98 | ST1 | NEV | | | |
| | | | 1/4/99 | ST2 | NEV | | | |
| | | | 2/4/99 | ST3 | PV1 | SABIN | 0.11 | |
| | | | 3/5/99 | ST4 | PV2 | SABIN | | |
| | | | 4/2/99 | ST5 | PV2 | SABIN | 0.22 | |
| | | | 4/30/99 | ST6 | NPEV | | | CA4 |

| Participant ID/age, months | HIV infection status | Routine OPV doses | Specimen Date | Specimen | Viral culture result | Genotype | Nucleotide changes, % | NPEV serotype |
|---|---|---|---|---|---|---|---|---|
| | | | 5/28/99 | ST7 | NPEV | | | CA24 |
| Q/14 | Uninf | Unk. | 9/16/98 | ST1 | NEGATIVE | | | |
| | | | 10/19/98 | ST2 | NEGATIVE | | | |
| | | | 11/2/98 | ST3 | NEGATIVE | | | |
| | | | 11/9/98 | ST4 | NEGATIVE | | | |
| | | | 12/3/98 | ST5 | NPEV | | | EV99 |
| | | | 12/8/98 | ST6 | NPEV | | | EV99 |
| | | | 1/4/99 | ST7 | NEGATIVE | | | |
| | | | 2/4/99 | ST8 | NEGATIVE | | | |
| | | | 3/5/99 | ST9 | NEGATIVE | | | |
| R/14 | Uninf | Unk. | 9/16/98 | ST1 | NPEV | | | E2 |
| | | | 10/19/98 | ST2 | NEGATIVE | | | |
| | | | 11/2/98 | ST3 | NEGATIVE | | | |
| | | | 11/9/98 | ST4 | NEGATIVE | | | |
| | | | 12/3/98 | ST5 | NPEV | | | E25 |
| | | | 12/8/98 | ST6 | NPEV | | | E25 |
| | | | 1/4/99 | ST7 | NEGATIVE | | | |
| | | | 2/4/99 | ST8 | NPEV | | | CA24 |
| | | | 3/5/99 | ST9 | NEGATIVE | | | |
| | | | 4/2/99 | ST10 | NEGATIVE | | | |
| | | | 4/30/99 | ST11 | NEGATIVE | | | |
| S/12 | Uninf | 3 doses, in 1997-1998 (last dose - 4/29/98) | 9/16/98 | ST1 | NEGATIVE | | | |
| | | | 10/19/98 | ST2 | NEGATIVE | | | |
| | | | 11/2/98 | ST3 | NEGATIVE | | | |
| | | | 11/9/98 | ST4 | NEGATIVE | | | |
| | | | 12/3/98 | ST5 | NPEV | | | E25 |
| | | | 12/8/98 | ST6 | NPEV | | | E25 |
| | | | 1/4/99 | ST7 | NPEV | | | E25 |
| | | | 2/4/99 | ST8 | NPEV | | | CA16 |
| | | | 3/5/99 | ST9 | NEGATIVE | | | |
| | | | 4/2/99 | ST10 | NEGATIVE | | | |
| | | | 4/30/99 | ST11 | NPEV | | | EV80 |
| | | | 5/28/99 | ST12 | NEGATIVE | | | |
| T/9 | Uninf | 4 doses, in 1998 (last dose - 11/11/98) | 1/4/99 | ST1 | NEV | | | |
| | | | 2/4/99 | ST2 | NEV | | | |
| | | | 3/5/99 | ST3 | NEGATIVE | | | |
| | | | 4/2/99 | ST4 | NEGATIVE | | | |
| | | | 4/30/99 | ST5 | NEGATIVE | | | |
| | | | 5/28/99 | ST6 | NPEV | | | CA24 |
| | | | 6/25/99 | ST7 | NPEV | | | E17 |
| | | | 7/22/99 | ST8 | NPEV | | | E4 |
| U/7 | Uninf | 3 doses, during | 2/4/99 | ST1 | NEV | | | |

| **Participant ID/age, months** | **HIV infection status** | **Routine OPV doses** | **Specimen Date** | **Specimen** | **Viral culture result** | **Genotype** | **Nucleotide changes, %** | **NPEV serotype** |
|---|---|---|---|---|---|---|---|---|
| | | enrolment (2/10/99, 3/10/99, 5/5/99) | 3/5/99 | ST2 | NEGATIVE | | | |
| | | | 4/2/99 | ST3 | PV3 | SABIN | | |
| | | | 4/30/99 | ST4 | NEGATIVE | | | |
| | | | 5/28/99 | ST5 | NEGATIVE | | | |
| | | | 6/25/99 | ST6 | NPEV | | | E17 |
| | | | 7/22/99 | ST7 | NPEV | | | E4 |
| | | | 8/18/99 | ST8 | NPEV | | | CA24 |

Inf., infected; Uninf., uninfected; ST, stool; NPEV, non-polio enterovirus; NEV, non-enterovirus (virus not characterized further); unk., unknown.

Sequences presented in Figure are underlined.
